# Evaluation of a dill (*Anethum graveolens* L.) gene bank germplasm collection using multivariate analysis of morphological traits, molecular genotyping and chemical composition to identify novel genotypes for plant breeding

**DOI:** 10.7717/peerj.15043

**Published:** 2023-03-29

**Authors:** Kalliopi Kadoglidou, Catherine Cook, Anastasia Boutsika, Eirini Sarrou, Ifigeneia Mellidou, Christina Aidonidou, Ioannis Grigoriadis, Andrea Angeli, Stefan Martens, Vasiliki Georgiadou, Theodoros Moysiadis, Parthenopi Ralli, Ioannis Mylonas, Nikolaos Tourvas, Michail Michailidis, Apostolos Kalivas, Eleni Maloupa, Ioannis Ganopoulos, Aliki Xanthopoulou

**Affiliations:** 1 ELGO DIMITRA, Institute of Plant Breeding and Genetic Resources, Thessaloniki, Greece; 2 Fondazione Edmund Mach, Center of Research and Innovation, San Michele all’Adige, Trento, Italy; 3 Department of Computer Science, School of Sciences and Engineering, University of Nicosia, Nicosia, Cyprus

**Keywords:** Dill, *Anethum graveolens*, Essential oil, Polyphenolics, Genetic diversity, Greek Gene Bank

## Abstract

Dill (*Anethum graveolens* L.) is an aromatic herb widely used in the food industry, with several commercial cultivars available with different qualitative characteristics. Commercial cultivars are usually preferred over landraces due to their higher yield and also the lack of improved landraces than can be commercialized. In Greece, however, traditional dill landraces are cultivated by local communities. Many are conserved in the Greek Gene Bank and the aim here was to investigate and compare the morphological, genetic, and chemical biodiversity of twenty-two Greek landraces and nine modern/commercial cultivars. Multivariate analysis of the morphological descriptors, molecular markers, and essential oil and polyphenol composition revealed that the Greek landraces were clearly distinguished compared with modern cultivars at the level of phenological, molecular and chemical traits. Landraces were typically taller, with larger umbels, denser foliage, and larger leaves. Plant height, density of foliage, density of feathering as well as aroma characteristics were desirable traits observed for some landraces, such as T538/06 and GRC-1348/04, which were similar or superior to those of some commercial cultivars. Polymorphic loci for inter-simple sequence repeat (ISSR) and start codon targeted (SCoT) molecular markers were 76.47% and 72.41% for landraces, and 68.24% and 43.10% for the modern cultivars, respectively. Genetic divergence was shown, but not complete isolation, indicating that some gene flow may have occurred between landraces and cultivars. The major constituent in all dill leaf essential oils was *α*-phellandrene (54.42–70.25%). Landraces had a higher *α*-phellandrene and dill ether content than cultivars. Two dill landraces were rich in chlorogenic acid, the main polyphenolic compound determined. The study highlighted for the first-time Greek landraces with desirable characteristics regarding quality, yield, and harvest time suitable for breeding programs to develop new dill cultivars with superior features.

## Introduction

Dill (*Anethum graveolens* L.) is an annual or biennial, herbaceous, seed-propagated plant that belongs to the family *Apiaceae* (*Umbelliferae*), and is the sole species in the genus *Anethum*. It is an upright, aromatic herb with feathery, segmented leaves with sheathed petioles. Flowers are yellow and located in umbels, which contain several peduncles. There is one main umbel and several smaller lateral umbels on branches from the main stem. Seeds are oval and flat with three longitudinal grooves and two wings (Jana & Shekhawat, 2012; Singh et al., 2020). Dill is a typical out-crossing species (Snell & Aarssen, 2005) and it is diploid (2*n* = 22) (Ma, Qin & Xing, 1984). This aromatic and medicinal plant is widely cultivated in Europe, the United States, and India (Suresh et al., 2013), while it is native to south-west Asia or south-east Europe (Bailer et al., 2001).

Leaves and seeds of dill are used for flavoring in culinary processes. As such, it is cultivated for the fresh or dried herb market, but high variability in morphological, aroma, and flavor characteristics are observed between cultivars. The green tissues and the fruits (seeds) contain essential oils, fatty oils, moisture, proteins, carbohydrates, fiber, ash, and mineral elements such as calcium, potassium, magnesium, phosphorous, sodium, vitamin A and niacin (Esmail Al-Snafi, 2014). Dill also contains other important phytonutrients, including furanocoumarin, 5-(4″-hydroxy-3″methyl-2″-butenyloxy)-6, 7-furocoumarin, oxypeucedanin, oxypeucedanin hydrate, falcarindiol and flavonoids (Ishikawa, Kudo & Kitajima, 2002; Radulescu, Popescu & Ilies, 2010; Stavri & Gibbons, 2005; Yazdanparast & Bahramikia, 2008). Dill leaf essential oil is rich in volatile compounds with *α*-phellandrene being the major compound, followed by *α*-pinene, *β*-phellandrene, dill ether, myristicin, dill apiole and apiole (Biesiada et al., 2019; Charles, Simon & Widrlechner, 1995; El-Zaeddi et al., 2020, 2017, 2016; Mohebodini & Kalalagh, 2021; Vokk et al., 2011). Leaf essential oil composition differs from that of seed essential oil which contains mainly carvone, but also dihydro-carvone and limonene (Charles, Simon & Widrlechner, 1995; Vokk et al., 2011). The biological properties of dill essential oil include antibacterial, antifungal, insecticidal, analgesic, and antioxidant activities, many of which are useful in food preservation (Biesiada et al., 2019; Kaur, Kaur & Bhardwaj, 2021; Vokk et al., 2011).

Several commercial dill cultivars for example, Ambrozja, Kronos, and Lukullus, are available with varying characteristics, such as, density and color of foliage, time of flowering, nutritional value, and intensity of aroma (UPOV, 1999; Biesiada et al., 2019). However, in Greece, traditional dill landraces are cultivated by local communities, being selected and adapted to the prevailing climatic and environmental conditions in which they are grown. Many of these landraces are conserved in the Greek Gene Bank but these have not yet been fully evaluated. Earlier studies on the phenological characterization of some Greek dill landraces demonstrated that morphological traits can efficiently group genotypes according to their geographical origin (Ninou et al., 2017). As a farther step, herein, the integration of phenotypic assessment and secondary metabolite diversity within Greek dill landraces could provide useful information for future breeding efforts. Molecular methods have been widely used to identify, and evaluate the genetic diversity within and among species of different aromatic and pharmaceutical plants (Solouki et al., 2008). DNA based markers are efficient tools which can be used to evaluate the genetic diversity and population structure of crop germplasm (Suresh et al., 2013). As far as we know, there is no survey in the literature coupling phenotypic, genetic and chemical diversity of dill landraces. However, few studies have been conducted so far, on either the phenotypic and the genetic, or the chemical fingerprint diversity of dill landraces and cultivars, primarily focusing on national dill collections (Charles, Simon & Widrlechner, 1995; Solouki et al., 2012; Suresh et al., 2013; Ninou et al., 2017; Sinhasane et al., 2022).

The aim of this study was to investigate and compare simultaneously the phenotypic, genetic, and chemical biodiversity of twenty-two Greek dill (*A. graveolens*) landraces from the Greek Gene Bank collection and nine modern cultivars for plants grown under identical conditions. Phenotypic diversity was determined for the main morphological characteristics using the UPOV descriptor list (UPOV, 1999), while inter-simple sequence repeat (ISSR) and start codon targeted (SCoT) molecular markers were used for assessing genetic diversity. The chemical diversity was evaluated using leaf essential oil composition and polyphenolic content with the aim of identifying qualitative target traits. To the best of our knowledge, this study represents the first comprehensive report of the genetic diversity of Greek dill landraces based on the morphological descriptors, molecular markers, and metabolites, and aims to identify promising dill germplasm which could be used in future breeding programs for the selection of superior and divergent genotypes.

## Materials and Methods

### Plant material

**Figure 1:**
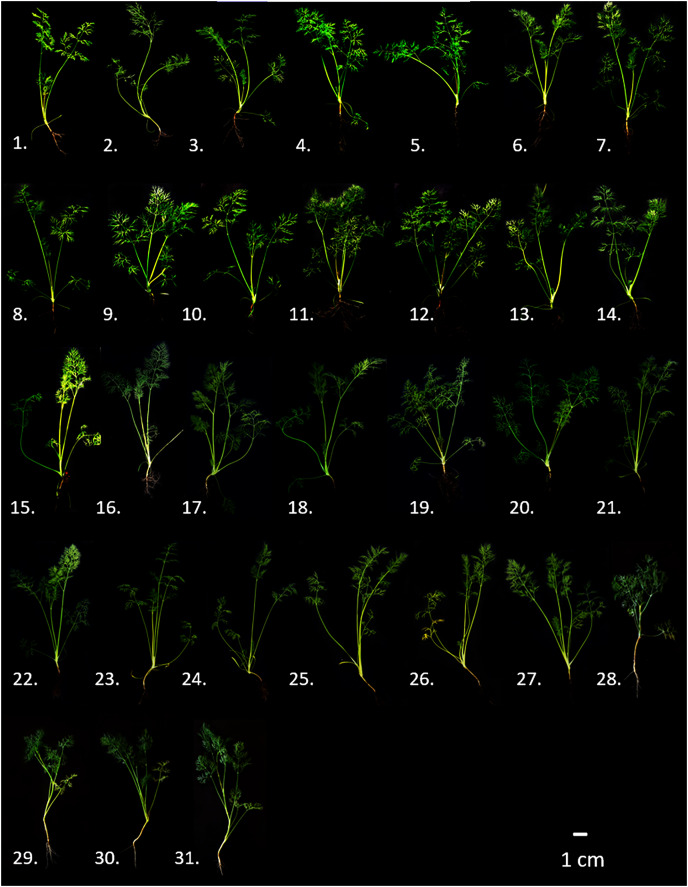
Comparative representation of indicative phenotypes of the thirty-one genotypes of dill (*A. graveolens*) studied. Landraces: 1. ‘T-518/06’, 2. ‘HL-232/07’, 3. ‘T-208/06’, 4. ‘T-538/06’, 5. ‘T-370/06’, 6. ‘T-349/06’, 7. ‘GRC-209/08’, 8. ‘GRC-1348/04’, 9. ‘IS-127/07’, 10. ‘T-326/06’, 11. ‘T-315/06’, 12. ‘T-309/06’, 13. ‘T-269/06’, 14. ‘KD-178/07’, 15. ‘CHA-035/07’, 16. ‘T-382/06’, 17. ‘ROX-064/07’, 18. ‘ANP-015/07’, 19. ‘T-399/06’, 20. ‘KD-235/07’, 21. ‘SAS-049/07’, 22. ‘K-133/06’; Cultivars: 23. ‘Szmaragd’, 24. ‘Diana’, 25. ‘Mariska’, 26. ‘Tetra’, 27. ‘Dukat’, 28. ‘Kronos’, 29. ‘Ambrozja’, 30. ‘Lukullus’, 31. ‘Amat’.

In total, thirty-one dill genotypes were examined: twenty-two landraces obtained from the Greek Gene Bank (Hellenic Agricultural Organization-Dimitra, Thessaloniki) and nine modern cultivars which were obtained commercially from Chiltern seeds (http://www.chilternseeds.co.uk) and gardenseedsmarket (https://gardenseedsmarket.com). Early in January 2021, in a greenhouse at the Institute of Plant Breeding and Genetic Resources, Thermi-Thessaloniki (40°32′08.7″N, 23°00′06.4″E), seeds of each genotype were sown in polystyrene containers using a mixture of peat and perlite as rooting media. Later, at the five to seven leaf-stage, thirty individual plants of each genotype were transplanted in net greenhouse isolation cages (40 cm within the row and 1 m inter-row distance). Each genotype was transplanted into a separate net greenhouse cage to prevent cross-pollination. Dill landrace (1–22) and modern cultivar plantlets (23–31) are shown in Fig. 1, whereas further relative information is given in Table S1.

### Phenotypic characterization and multivariate analysis

Phenotypic characterization was carried out based on twenty-two morphological descriptors, involving nominal and ordinal measurements according to UPOV (1999) (Data S1). The traits recorded for young plants were the anthocyanin coloration (YPAC) and the attitude of leaves (YPAL). For the whole plant, the recorded traits were the density of foliage (PDF), the number of primary branches (PNPB), the height (PH) and the length of the main stem (PLMS). For the stem, the diameter (SD), the blue hue (SBH), the intensity of green color (SIG) and the waxiness (SW) were recorded. With respect to the leaves, the shape (LS), the density of feathering (LDF), the width of segments (LWS), the length (LL) and the width (LW), the blue hue (LBH), the intensity of green color (LIG) and the waxiness (LW) were recorded. Finally, observations on the umbel included the diameter (UD), the number of peduncles (UNP), the time of appearance of main umbel (UTAMU) and the time of the beginning of flowering (UTBF). Tests and methods were applied and conducted according to the UPOV guidelines.

Non-parametric Spearman correlations were used for the determination of the relations between the morphological traits (Mellidou et al., 2020). Principal component analysis (PCA), which provides the multivariate statistical analysis of a dataset, was used to identify the main traits included in the dataset obtained. Multivariate PCA in the XLSTAT software (2019) was used to categorize genotypes based on both nominal and ordinal morphological features (2019). PCA and agglomerative hierarchical clustering (AHC) were generated using the web tool for visualizing clustering of multivariate data ‘ClustVis’ (https://biit.cs.ut.ee/clustvis), while the construction of two-dimensional (2-D) plots was based on the first two principal components (PCs). The AHC analysis was performed using Euclidean distance and Ward’s method for agglomeration to systematically analyze combined evaluated morphological traits per dill genotype.

### Genetic characterization using inter-simple sequence repeat (ISSR) and start codon targeted (SCoT) molecular markers

DNA was isolated from young leaves using the NucleoSpin® Plant II Kit (Macherey-Nagel, Nordrhein-Westfalen, Germany) according to the manufacturer’s instructions. DNA concentration and quality were estimated spectrophotometrically at 260 and 280 nm using an Eppendorf BioPhotometer (Eppendorf, Hamburg, Germany). The integrity of the DNA was determined using gel electrophoresis on a 0.8% (w/v) agarose gel.

After preliminary screening of 20 ISSR (University of British Columbia—UBC) and ScoT primers (Collard & Mackill, 2009), 12 unambiguously scorable and reproducible markers were selected (six ISSRs and six SCoTs) based on Rp, MI and PIC values. Oligonucleotide primers complementary to simple sequence repeats (UBC807, UBC810, UBC811, UBC834, UBC840, UBC860) and others complementary to codon targeted polymorphisms (SCoT1, SCoT13, SCoT33, SCoT34, SCoT51, SCoT61) were used for PCR amplification. The reaction mixture (total volume 25 μL) contained the following reagents: 0.5 μL dNTPs (10 mM), 2.5 μL 10×-buffer, 1 μL primer (10 mM), 0.5 μL template DNA, 0.1 μL Taq polymerase (5 U/μL) and 20.3 μL sterile dd H_2_O. PCR amplifications were carried out according to Stathi et al. (2020), using an Eppendorf Mastercycler EP Thermal Cycler Range (Eppendorf, Hamburg, Germany): DNA denaturation was initiated at 95 °C for 5 min, followed by 35 cycles at 95 °C of 30 s, for DNA annealing T at 48–56 °C (all at 52 °C except for UBC811 at 48 °C), for 90 s for annealing of the primers and at 72 °C for 90 s for chain extension. The temperature was held at 72 °C for 5 min after the 35 cycles were completed.

The amplification products of the ISSR and SCoTs markers were separated by electrophoresis on a 1.5% (w/v) agarose gel and stained with ethidium bromide. The size marker was a 2-log DNA ladder (New England Biolabs, Ipswich, MA, USA). Gels were exposed to UV light in a UVItec Transilluminator (UVItec Limited, Cambridge, UK), and UVIDoc software UVIDocMw version 99.04 (UVItec Limited, Cambridge, UK) was used for analysis.

Using the Genemapper v4.0 software and an internal standard (GS 500 LIZ; Applied Biosystems, Waltham, MA, USA), the size of the detected fragments was determined. To reduce the impact of potential size homoplasy, fragments ranging in size from 150 to 500 bases were counted and analyzed further. ISSRs and SCoTs alleles were scored based on whether specific fragments were present (1) or absent (0). The phenotypic data matrices for genetic (ISSR + SCoT) information were also generated. All matrices were subsequently analyzed identically. The performance of the primers was measured by calculating different parameters including polymorphic information content (PIC), resolving power (RP), and marker index (MI) for each primer by the program iMEC (Amiryousefi, Hyvönen & Poczai, 2018). These parameters described by Prevost & Wilkinson (1999), Munda et al. (2022) and Lal et al. (2022). Nei’s coefficient (Lynch & Milligan, 1994) was determined to assess the genetic variance within the groups of genotypes, while Nei’s formula was used to determine genetic distance (Nei, 1978). PCA was used as a graphical representation of a matrix to show how closely related the landraces and cultivars were. Analyses were carried out using GenAlex 6.5 (Peakall & Smouse, 2012) and Microsoft® Excel 2010/XLSTAT©-Pro software (Version 2013.4.07; Addinsoft Inc., Brooklyn, NY, USA).

The Dice similarity coefficient (Dice, 1945) as implemented in the ade4 1.7–15 R package (Dray & Dufour, 2007) was used along with the UPGMA clustering algorithm to perform cluster analysis in R 4.0.2 (R Core Team, 2020). The ape 5.4–1 package was used to compute bootstrap support (1,000 bootstraps), and the phytools 0.7–47 package was used to display the resulting dendrograms (Revell, 2012). The ‘admixture’ and ‘independent allele frequencies’ models were used to run STRUCTURE 2.3.4 (Pritchard, Stephens & Donnelly, 2000). With a burn-in of 500,000 iterations and 1,000,000 MCMC repetitions for each run, 20 replicates from K = 1 to K = 6 were used. K was inferred using Evanno’s method (Evanno, Regnaut & Goudet, 2005), which was run in the pophelper 2.3.0 R package (Francis, 2017). The software Structure threader (Pina-Martins et al., 2017) was used to parallelize distinct runs of K.

### Extraction of polyphenolic compounds and ultra performance liquid chromatography-mass spectrometry (LC-MS/MS, MRM analyses)

Fresh, green leaf tissues from each dill genotype were collected in the vegetative stage and freeze-dried at −24 °C (Freeze-dryer Alpha 1–2 LD plus; Christ, Osterode, Germany). Samples were ground to a fine powder using a laboratory grinder mill, IKA A11 (IKA-Werke, Staufen, Germany). The extraction of polyphenolic compounds and LC-MS/MS analysis was accomplished as previously reported Boutsika et al. (2021). Three independent replicates were employed for each dill genotype.

Targeted UPLC analysis was performed on a Waters Acquity UPLC system (Milford, MA, USA) and separation of the phenolic compounds was carried out using a Waters Acquity HSS T3 column (1.8 μm, 100 mm × 2.1 mm), at 40 °C. Phenolic compounds were analyzed as described by Vrhovsek et al. (2012) using water and acetonitrile as mobile phases for the gradient. A Waters Xevo TQMS system equipped with an electrospray (ESI) source was used for mass spectrometry detection. Data was processed using the Mass Lynx Target Lynx Application Manager (Waters).

### Essential oil isolation and analyses

Fresh leaves with stems (petioles) were cut at the base of plants of each dill genotype in the vegetative stage, in early March. Samples of 100–120 g fresh weight (FW) were cut coarsely with care into sections with a length of 2–5 cm and were then hydro-distilled for 3 h using a Clevenger-type apparatus. The essential oil recovered was measured (v) and stored at −10 °C in the dark. The essential oil yield (mL 100 g^−1^ FW) was expressed as % (v/w).

Essential oil analysis was carried out using a gas chromatography system as previously described with the relative chromatographic conditions in Boutsika et al. (2021). Specifically, a TRACE™ Gas Chromatograph (GC) with a combination of either a flame ionization detector (FID) or a mass spectrometer (MS) (PolarisQ; Thermo Scientific, Waltham, MA, USA, formerly Thermo Finnigan, San Jose, CA, USA) was used.

Additionally, equipment control, data acquisition and chromatogram analysis were performed as previously described in Boutsika et al. (2021) by using the software packages ChromQuest V4.0 and the Xcalibur™ (Thermo Scientific, Waltham, MA, USA) for GC-FID and GC-MS, respectively. Specifically, comparisons of experimental and known mass spectra were conducted (Adams, 2007; Mass Spectrometry Data Center, NIST, n.d., https://chemdata.nist.gov/; McLafferty, 2008). According to Adams (2007), the retention times of the essential oil constituents and a homologous series of C_8_–C_26_ n-alkane standards were used for the determination of the arithmetic indices (AI) and Kovats indices (KI) (Chiron AS, Trondheim, Norway).

### Correlation network of secondary metabolites

The correlation of the essential oil components and the polyphenolic compounds was assessed based on the Pearson coefficient. Firstly, a correlation analysis was performed for all pairwise comparisons. Next, a network analysis was used, in which the essential oil components and polyphenols were represented by nodes, and all statistically significant correlations between the nodes were represented by edges.

## Results

### Phenotypic characterization and diversity between landraces and cultivars

The study of plant morphological traits represents a quick and useful tool for the preliminary evaluation of the extent of genetic diversity related to the phenotype (Solouki et al., 2012). Among the most common statistical indicators to assess the genetic diversity of traits related to the phenotype are the mean, the standard deviation, and the coefficient of variance (CV%). The set of twenty-two morphological descriptors used to evaluate the twenty-two landraces from the Greek Gene Bank collection and the nine cultivars are given in Data S1.

Most of the selected descriptors were polymorphic exhibiting more than two phenotypes, except for YPAC, SBH and LBH, which had only two categories (absent and present, Data S1). Among the polymorphic traits, YPAL, LS, LW, LWAX, UTAMU and UTBF exhibited the highest CV%, with 67.48%, 32.50%, 29.41%, 34.78%, 30.40% and 35.19%, respectively (data not shown). On the contrary, the traits UD, UNPI and SIG presented the lowest CV% (below 12%), indicating that these are less informative, at least for the genotypes studied herein. Spearman correlation analysis was used to evaluate the relationship between the morphological descriptors (Data S2). Significant, positive correlations (*p* ≤ 0.001) were observed between some traits, indicating that when selection or breeding is applied for one of these traits, an indirect improvement could also be observed in other traits (Irakli et al., 2021). Indicatively, PH was strongly correlated with PLMS (0.778) and UTAMU with UTBF (0.748), whereas PNPB was moderately correlated with SD (0.564), PLMS with LS (0.534) and SIG with LBH (0.525). Nevertheless, LBH was strongly, negatively correlated with PH (−0.631) and PLMS (−0.631) and moderately, negatively correlated with PNPB (−0.533) and LS (−0.594). Also, LS and YPAL were moderately, negatively correlated (−0.512).

**Figure 2:**
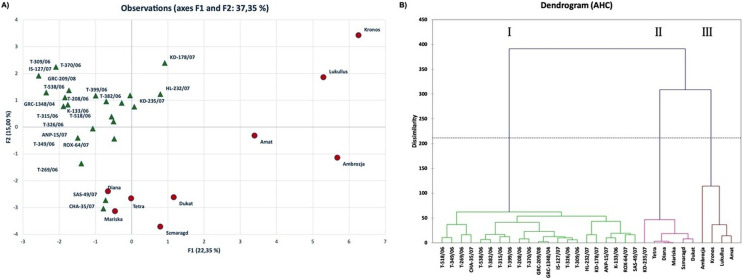
Multivariate analysis based on twenty-two morphological traits evaluated for thirty-one dill (*A. graveolens*) accessions studied. (A) Two-D PCA plot of the first two components obtained from principal component analysis (PCA) and (B) dendrogram from agglomerative hierarchical clustering (AHC) analysis.

PCA allowed the identification of the most important morphological traits which discriminated the genotypes studied herein. The distribution of genotypes based on PC1 and PC2, showed part of the underlying phenotypic variation among them (37.35%) (Fig. 2A). Kaiser’s criterion (Eigenvalue >1) (Kaiser, 1958) was satisfied by five components (total variation accounted for 61.36%, Table S2). The first component, accounting for 22.35% of the total variation, included the traits PNPB, PH, PLMS, SIG, LS, LBH and LWAX, while the second component included YPAC, LS, LWS, LW and LIG. Traits such as SD and UD were the most important ones contributing to the third component, whereas YPAL and LDF were the major determinants of components four and five, respectively.

Interestingly, most of the native Greek dill genotypes were distinctly ordinated to the negative side of PC1 in both the upper and lower quadrants of PC2 (Fig. 2A). On the contrary, six of the nine modern cultivars were separated from the Greek genotypes and were grouped on the positive side of PC1 in the upper and lower quadrants of PC2. Thus, the Greek landraces were separated from the modern cultivars ‘Kronos’, ‘Lukullus’, ‘Amat’, ‘Ambrozja’, ‘Dukat’ and ‘Szmaragd’ based on their morphological characteristics.

Agglomerative hierarchical clustering (AHC, dendrogram) analysis was used to enable grouping of genotypes into clusters of similar responses based on calculations of Euclidean distance, to acquire a thorough overview of the distribution of dill genotypes based on the morphological traits (Fig. 2B). The output dendrogram obtained after applying Ward’s method for agglomeration, revealed three distinct groups: group I comprised of 21 of the 22 Greek landraces, whereas groups II and III consisted of the modern cultivars and the Greek landrace ‘KD-235/07’. More specifically, AHC analysis grouped in Cluster I all the genotypes from the Greek Gene Bank, except for ‘KD-235/07’. Cluster I was distinctly separated from the modern cultivars, which were distributed in clusters II and III. Specifically, four modern cultivars, ‘Ambrozja’, ‘Kronos’, ‘Lukullus’ and ‘Amat’, grouped in cluster III, were very distant from the Greek landraces revealing differences in most traits evaluated in this study.

### Genetic characterization and diversity between landraces and cultivars

Agarose gel of the PCR reactions using UBC834, UBC811 and SCOT33 primers are depicted in Fig. S1. The PIC values of the ISSR primers ranged from 0.281 (UBC811) to 0.345 (UBC834), with an average of 0.306. The MI and Rp values ranged from 0.289 (UBC811) to 0.396 (UBC834), and from 4.932 (UBC811) to 7.832 (UBC834), with average values of 0.344 and 6.349 per ISSR primer, respectively. Additionally, for SCoT primers, the PIC values ranged from 0.092 (ScoT51) to 0.331 (ScoT13) (Table S3). MI and Rp were 0.339 and 7.654, respectively, for ScoT13 primer, while the lowest MI and Rp were exhibited by SCoT51 primer (0.101 and 4.747, respectively). The average MI and Rp for all primers were 0.227 and 6.071, respectively.

Using ISSR molecular markers, a total of 85 fragments in the length range of 450–4,000 bp were detected, with similar numbers detected for landraces (78 bands, with 10 private bands) and cultivars (75 bands, with seven private bands). Landraces had a larger percentage of polymorphic loci (76.47%) than cultivars (68.24%). In contrast, the value of Nei’s gene diversity was higher for the cultivars (0.262) than the landraces (0.256), while Shannon’s Index (I) was 0.386 for landraces and 0.385 for cultivars (Table S4).

Using SCoT molecular markers, a total of 58 fragments in the length range of 450–4,000 bp were detected with higher numbers detected for landraces (57 bands, with nine private bands) than in cultivars (49 bands, with one private band). Landraces had a larger percentage of polymorphic loci (72.41%) than cultivars (43.10%). The value of Nei’s gene diversity was also higher for the landraces (0.199) than the cultivars (0.167), and Shannon’s Index (I) was 0.313 for landraces and 0.245 for cultivars (Table S4).

Nei’s genetic distance exhibited a moderate value of 0.111 along with ΦST = 0.169 (*p* ≤ 0.001, 999 permutations) for the ISSR markers, whereas lower differentiation levels were observed for SCoT markers, with a Nei’s genetic distance of 0.057 and ΦST = 0.128 (*p* ≤ 0.001, 999 permutations).

**Figure 3:**
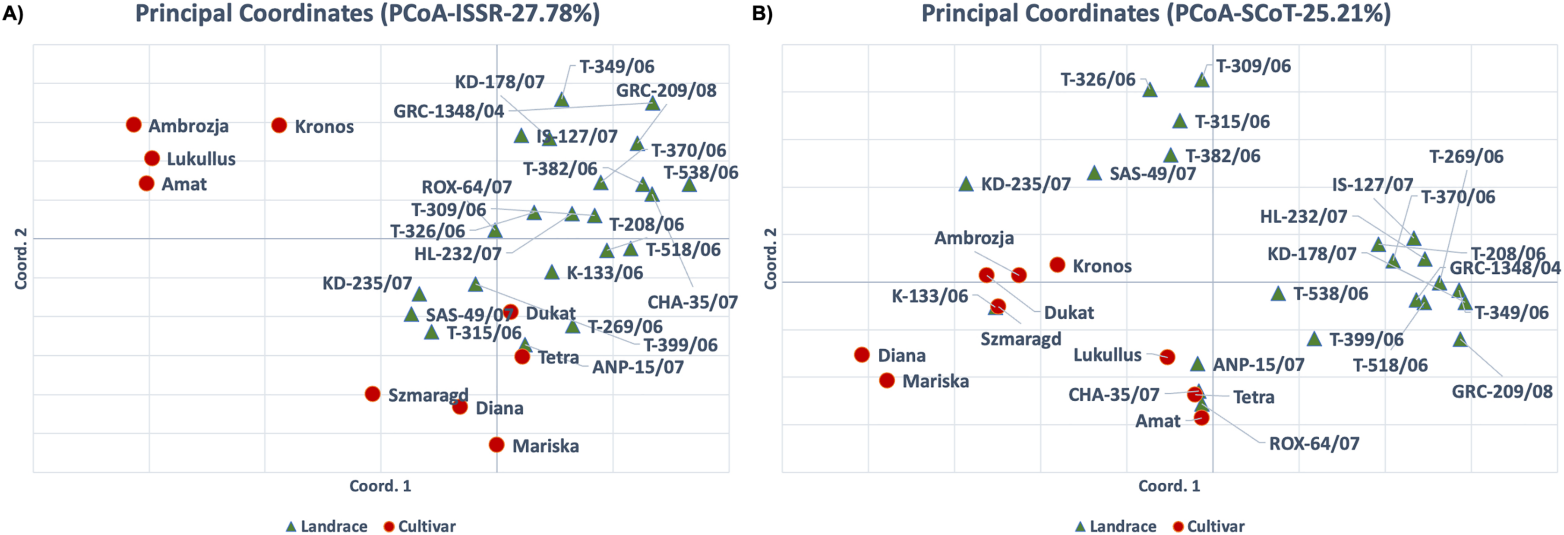
Principal component analysis (PCA) for thirty-one dill (*A. graveolens*) genotypes (22 Greek landraces and nine cultivars) using datasets for (A) ISSR and (B) SCoT molecular markers.

For the ISSR molecular marker data set, the PCA analysis results were consistent with the cluster analysis, and the first two axes accounted for 27.78% of the total variation (Fig. 3A). The cultivars ‘Kronos’, ‘Ambrozja’, ‘Lukullus’ and ‘Amat’ were in the upper left quadrant of the figure, whereas the majority of genotypes were found in the lower and upper right quadrants. The landraces ‘T-315/06’, ‘SAS-49/07’, ‘KD-235/07’ and ‘T-399/06’, and the cultivars ‘Szmaragd’ and ‘Diana’, formed a distinct group in the center of the plot. For the PCA analysis of the SCoT molecular marker data set, the first two axes accounted for 26.73% of the total variation, and the samples were generally distributed throughout the figure, with only ‘Amat’ and ‘T-518/06’ forming a separate cluster (Fig. 3B).

**Figure 4:**
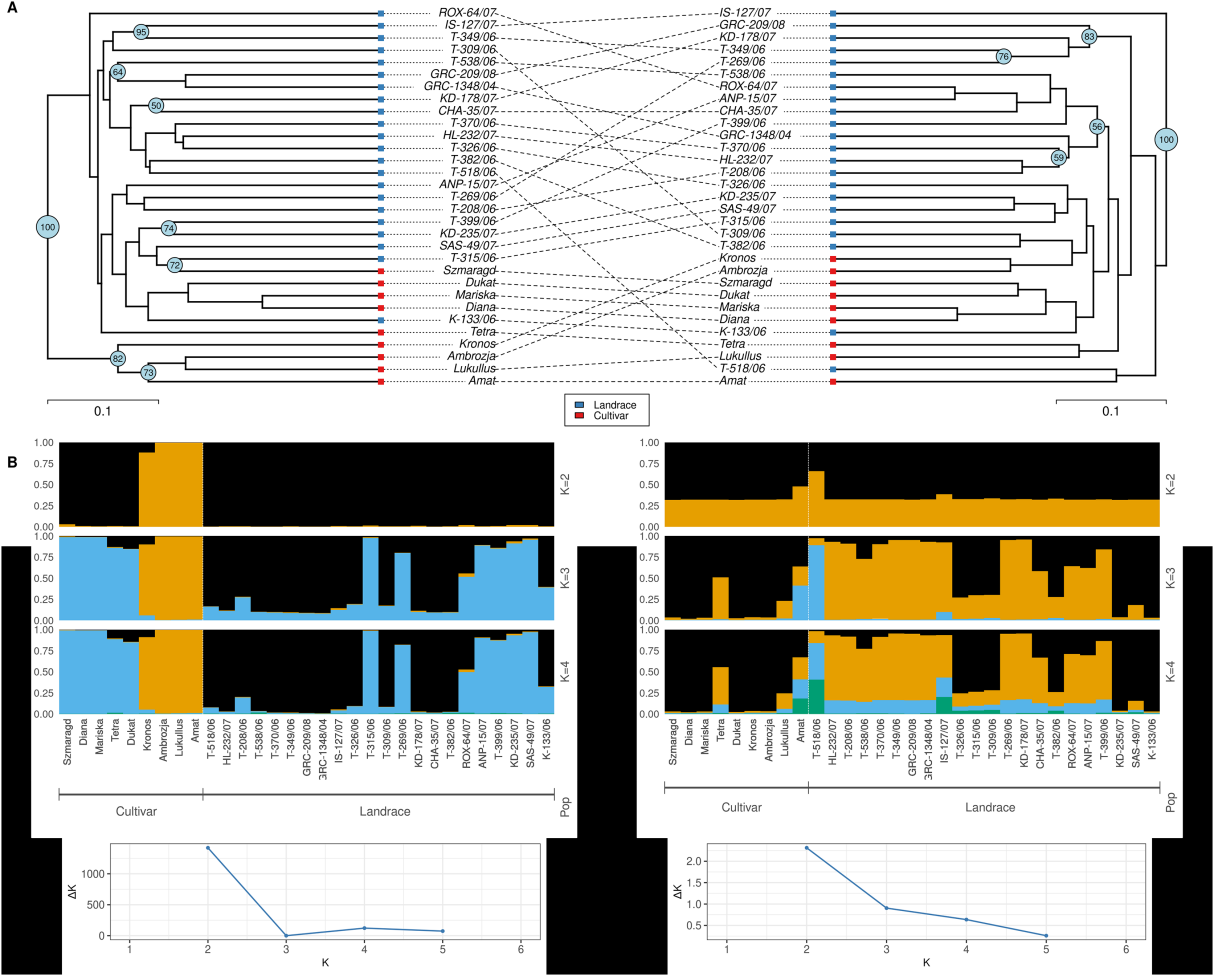
Cluster analysis and Bayesian assignment into groups of thirty-one dill (*A. graveolens*) genotypes (22 Greek landraces and nine cultivars) based on results for the molecular markers: ISSR data set (left) and SCoT data set (right). (A) UPGMA dendrogram based on Dice distance. (B) Group assignment for K = 2 up to K = 4. (C) Evanno’s ΔK statistic.

Dice distance based UPGMA dendrograms were constructed for both ISSR and SCoT molecular markers (Fig. 4A) and in both cases, three clusters were identified. For the ISSR data set, Cluster I contained the landrace ‘ROX-64/07’, Cluster II contained the cultivars ‘Tetra’, ‘Kronos’, ‘Ambrosia’, ‘Lukullus’, ‘Amat’, while Cluster III contained the remaining genotypes. For the SCoT data set, a slightly different pattern was observed, Cluster I contained the landrace ‘IS-127/07’, Cluster II contained the landrace ‘T-518/06’ and the cultivar ‘Amat’, while Cluster III contained the remaining genotypes.

Additionally, an in-depth STRUCTURE analysis was carried out. For the ISSR molecular marker’s dataset a value of K = 2 was optimal for Evanno’s ΔK statistic (Evanno, Regnaut & Goudet, 2005). Except for cultivars ‘Kronos’, ‘Ambrozja’, ‘Lukullus’ and ‘Amat’, all genotypes were grouped together (Fig. 4B). For the SCoT molecular marker’s dataset, Evanno’s ad-hoc statistic K also suggested a solution of K = 2 even though the ΔK values were significantly lower than for ISSRs, indicating a faint signal. Except for ‘Amat’ and ‘T-518/06’, all genotypes were loosely clustered in one group for K = 2 (Fig. 4C).

### Polyphenolic content

The hydro-methanolic extracts of the green, leaf tissues of dill samples were analyzed using a targeted UHPLC-MS/MS method to identify the quantitative polyphenolic profile of the 31 genotypes (local landraces and modern cultivars). The analysis identified the presence of three main phenolic metabolites: chlorogenic acid (*syn*. 3-*O*-caffeoylquinic acid), neo-chlorogenic acid (*syn*. 5-*O*-caffeoylquinic acid) and quercetin-3-*O*-glucuronide. These findings are consistent with previous reports on *A. graveolens* leaf extracts (El-Zaeddi et al., 2017; Wasli et al., 2018). Chlorogenic acid was the main polyphenolic compound which ranged from 2.035 mg 100 g^−1^ FW (‘KD-178/07’) to 10.350 mg 100 g^−1^ FW (‘Tetra’), followed by quercetin-3-*O*-glucuronide which ranged from 0.0035 mg 100 g^−1^ FW (‘T-208/06’) to 0.4351 mg 100 g^−1^ FW (‘Tetra’), and neo-chlorogenic acid which was only detected in trace amounts (0.00–0.0032 mg 100 g^−1^ FW) in the studied genotypes (Table S5). It is noteworthy that the Greek local landraces ‘T-538/06’ and ‘GRC-1348/04’ had high amounts of chlorogenic acid (7.972 and 7.997 mg 100 g^−1^ FW, respectively), similar to those of the modern cultivars ‘Szmaragd’, ‘Ambrozja’, ‘Diana’ and ‘Mariska’ (6.748–8.673 mg 100 g^−1^ FW) (Table S5).

### Essential oil content and composition

The essential oil yield of fresh dill leaves (leaves and petioles) ranged from 0.012% (‘GRC-209/08’) to 0.057% (‘HL-232/07’) for the twenty-two Greek landraces, whereas it ranged from 0.02% (‘Dukat’) to 0.041% (‘Diana’) for five of the modern cultivars (Table S5). The essential oil yields of four landraces (0.045% for ‘SAS-049/07’; 0.04% for ‘ROX-064/07’; 0.049% for ‘T-382/06’; 0.057% for ‘HL-232/07’) were equal to or greater than the highest yielding commercial cultivar ‘Diana’.

A total of twenty-one constituents, with a percentage composition of more than 0.1% in at least one dill essential oil, were identified in the thirty-one-dill leaf essential oils examined (Table S5). The main constituents in these thirty-one essential oils were *α*--pinene (0.99–2.33%), *α*-phellandrene (54.42–70.25%), *β*-phellandrene (9.38–12.35%), dill ether (0.62–22.22%), myristicin (0–18.56%), and dill apiole (0–11.12%) (Table S5). These constituents were present in most of the 31 dill leaf essential oils, but the percentage composition differed between dill genotypes, particularly with respect to the content of dill ether, myristicin and dill apiole.

Of the 22 gene bank landraces, nineteen had very similar essential oil composition, that is, *α*-pinene (1.07–1.38%), *α*-phellandrene (61.16–70.25%), *β*-phellandrene (10.62–12.35%), dill ether (4.8–22.22%), myristicin (0–7.81%), and dill apiole (0.01–5.88%) (Table S5), while the other three landraces ‘ROX-064/07’, ‘ANP-015/07’, and ‘SAS-049/07’ had a content of *α*-pinene (1.35–1.38%), *α*-phellandrene (64.43–67.27%), *β*-phellandrene (10.7–11.46%), dill ether (4.48–7.93%), myristicin (7.11–10.82%), and dill apiole (0–3.86%), differing with respect to a lower dill ether content and higher myristicin content, and were intermediary between the 19 landraces and the modern cultivars.

In contrast, the essential oil composition of five modern cultivars (‘Diana’, ‘Dukat’, ‘Mariska’, ‘Szmaragd’, ‘Tetra’) ranged from 0.99% to 1.21% for *α*-pinene, from 54.42% to 62.31% for *α*-phellandrene, from 9.38% to 10.95% for *β*-phellandrene, from 0.62% to 2.69% for dill ether, from 13.6% to18.56% for myristicin, and from 2.44% to 11.12% for dill apiole. Interestingly, modern cultivars had lower *α*-phellandrene and dill ether contents, as well as higher myristicin and dill apiole contents compared to the landraces. This is supported by the strongly, negative, correlation value (−0.895) observed for the pairwise comparison of dill ether and myristicin, using Pearson’s correlation analysis. The other four modern cultivars (‘Amat’, ‘Ambrozja’, ‘Kronos’, ‘Lukullus’) had essential oil compositions in a range of 2.22–2.33% for*α*-pinene, 59.95–63.86% for *α*-phellandrene, 11.88–12.08% for *β*-phellandrene, 4.61–12.13% for dill ether, 4.2–14.39% for myristicin, and 0.15–2.23% for dill apiole. In this regard, they had higher *α*-pinene and dill ether contents, as well as lower myristicin and dill apiole contents, compared to the other modern cultivars.

**Figure 5:**
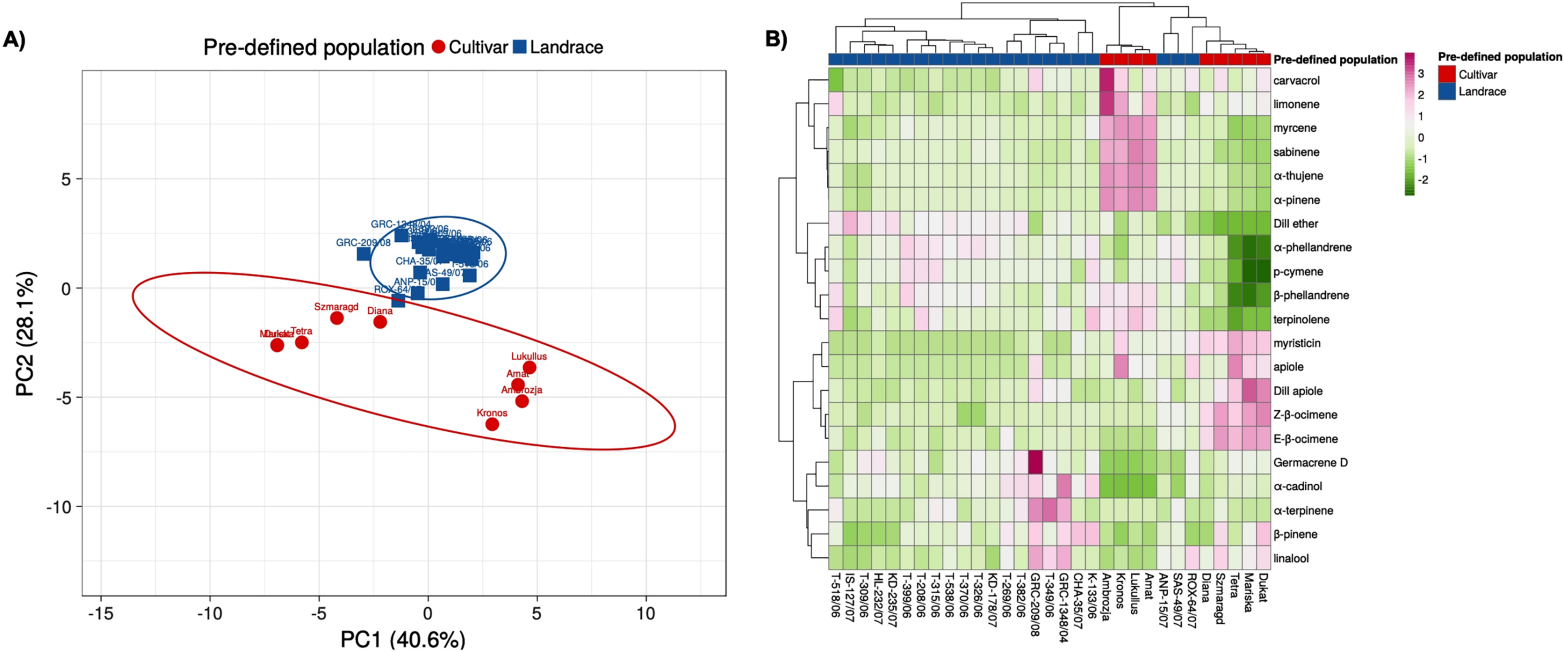
Multivariate analysis based on the leaf essential oil composition (21 volatile compounds) and three polyphenolic compounds of the 31 dill (*A. graveolens*) accessions studied. (A) Two-D PCA plot of the first two components obtained from principal component analysis (PCA) showing discriminated genotypes. (B) Heatmap representing the qualitative and quantitative essential oil and polyphenolic profile of each dill accession. Mean log values of the essential oil volatile compounds and the polyphenolic compounds are displayed using a color scale from green (minimum) to purple (maximum). The pre-defined populations are landraces (blue), and cultivars (red).

Principal component analysis based on twenty-one essential oil constituents and three polyphenolic compounds present in the collection, resulted in the identification of two main groups: one containing the gene bank landraces and the other the modern cultivars (Fig. 5A). The gene bank landraces were quite homogeneous in essential oil composition and polyphenolic compounds except for three landraces (‘ROX-064/07’, ‘ANP-015/07’, ‘SAS-049/07’). The cultivars ‘Mariska’ and ‘Dukat’ had almost identical essential oil composition, while there appeared to be two subsets within the cultivar group. The PC axis 1 accounted for 37.3% of the variation and axis 2 for 28.1%, with a combined variance of 65.4%. Similar clustering (three main clusters) based on essential oil and polyphenolic composition of the 31 landraces and cultivars was observed in the heatmap analysis with the three landraces ‘ROX-064/07’, ‘ANP-015/07’, ‘SAS-049/07’ clustering more closely with the cultivars ‘Diana’, ‘Dukat’, ‘Mariska’, ‘Szmaragd’, and ‘Tetra’ (Fig. 5B; Table S5).

### Correlation network analysis

**Figure 6:**
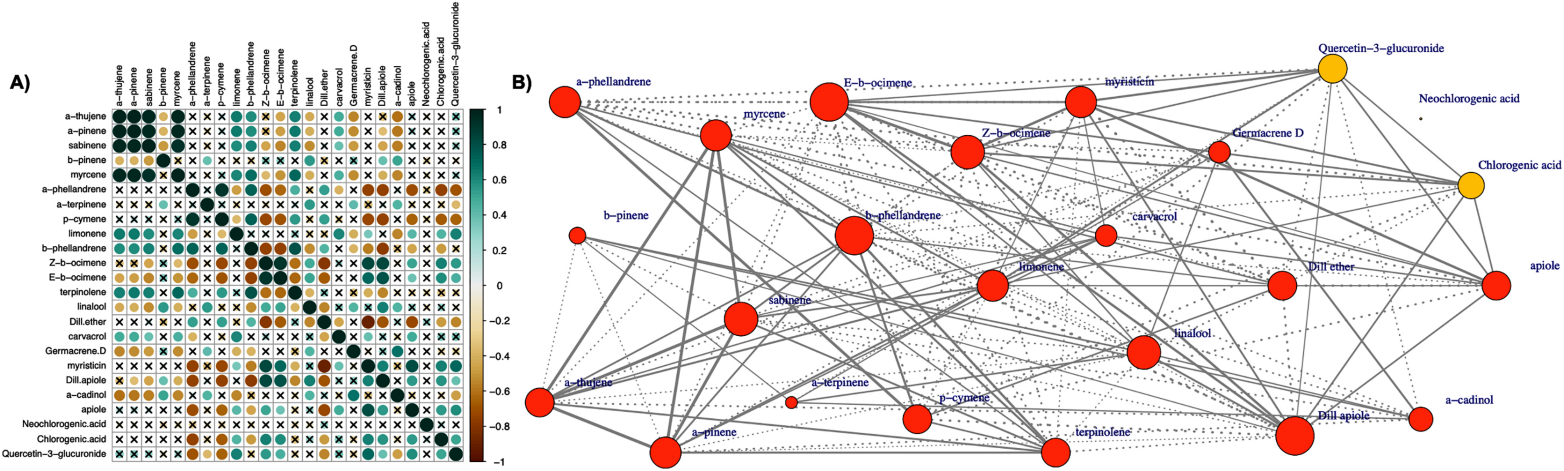
(A) Correlation analysis (Pearson coefficient) of all pairwise comparisons between 21 essential oil components and three polyphenolic compounds determined in leaves of the 31 dill (*A. graveolens*) accessions studied. The magnitude of the correlation is depicted in both the color and size of the spheres. Correlations which were not statistically significant (*p* value > 0.05) are marked with an ‘x’. (B) Correlation network of 21 essential oil components and three polyphenolic compounds (orange and yellow, respectively). The edges connecting the nodes are displayed only when the nodes are statistically significantly correlated (Pearson coefficient, *p* value = 0.05). Solid lines correspond to positive correlations and dotted lines correspond to negative correlations. The thickness of the lines reflects the magnitude of the correlation (absolute values). The size of the node indicates the degree of centrality (number of edges drawn from the node).

The Pearson correlation analysis (Fig. 6A) referred to all pairwise comparisons of essential oil components and the main polyphenolic compounds resulted in 276 pairwise comparisons in total, of which 138 (50%) were positive correlations and 138 (50%) were negative correlations. The maximum positive correlation was 0.997 and was observed for the comparison of *‘α*-pinene’ and ‘*α*-thujene’. The minimum negative correlation observed was −0.895 corresponding to the comparison between ‘myristicin’ and ‘dill ether’. Of the 138 positive and 138 negative correlations, 72 (52.17%) and 67 (48.55%), respectively, were statistically significant at 5% level of significance. Except for neo-chlorogenic acid, all other compounds exhibited both positive and negative statistically significant correlations with the remaining metabolites. Neo-chlorogenic acid did not exhibit any statistically significant correlation, suggesting that its metabolic pathway is not tightly co-regulated along with the other flavonoids.

In the corresponding correlation network (Fig. 6B), the edges between nodes correspond to statistically significant correlations, and their thickness represents the absolute value of the correlation. The size of the node indicates the number of edges drawn from the node (degree of centrality). The mean degree of centrality was found to be overall 11.58 (12.14 and 7.67 for essential oil compounds and polyphenols, respectively), with the maximum value 16 observed three times (‘*β*-phellandrene’, ‘(E)-*β*-ocimene’, and ‘dill apiole’). The minimum degree of centrality was observed in the case of ‘neo-chlorogenic acid’ with a minimum value of zero.

## Discussion

### Phenotypic diversity between landraces and cultivars

Overall, it was found that the main characteristics that distinguished between the landraces and cultivars were plant height, the presence of pigmentation, the shape and size of the leaves, the timing of the appearance of the main umbel, and the timing of the start of flowering (Data S1). Almost all landraces (more than 19 of 22) were classified as ‘tall’, with a ‘long’ main stem, ‘large’ main umbel diameter, ‘many’ peduncles in the umbel, and mainly ‘rhomboidal’ leaf shape. Additionally, most landraces (more than 14 out of 22 genotypes) had an erect attitude of leaves, medium to dense density of foliage (15 of 22) and density of leaf feathering (20 of 22), medium to broad leaf length (20 of 22) and width (19 of 22), as well medium to broad width of leaf segments (22 of 22) (Data S1). Most of the aforementioned morphological characteristics of Greek dill landraces are very important as they have a direct effect on the yield (fresh weight) (Said-Al Ahl & Omer, 2016; Karkleliene et al., 2014). Further, the Greek dill landraces ‘T-349/06’ and ‘T-326/06’ were distinguished by ‘large’ main umbel diameter, ‘early’ time of appearance of main umbel and ‘early’ time of beginning of flowering, which is in accordance with a previous study by Ninou et al. (2017). These morphological traits are desirable and–in contrast with the modern cultivars–they were observed only in the Greek landraces, suggesting their great potential in crop improvement. Specifically, the traits ‘early time of appearance of main umbel’ and ‘early time of beginning of flowering’ can contribute to the earlier harvesting potential.

### Genetic diversity between landraces and cultivars

Understanding molecular diversity (identification of individuals, populations, varieties, or breeds) is essential for the preservation of germplasm (Duran et al., 2009). Molecular methods can be used to locate alleles that may affect an organism’s capacity to endure in various environments. Many molecular markers have been used to detect DNA polymorphism and estimate the genetic relationships within and between species (Marsjan & Oldenbroek, 2007). The main advantage of molecular markers is their absence of bias, which means that they are unaffected by factors such as age and environment (Rahimi et al., 2018; Samantaray, Dhagat & Maiti, 2010).

Several studies have been carried out for the identification and classification of different species using molecular markers. For members of the *Apiaceae* family, random amplified polymorphic DNA (RAPD) and inter simple sequence repeats (ISSR) have been successfully used in the recent years for *Changium smyrnioides* (Fu, Qiu & Kong, 2003), *Achillea millefolium* (Farajpour et al., 2012), dill (Solouki et al., 2012; Suresh et al., 2013), cumin (Choudhary et al., 2015), coriander (Choudhary et al., 2019), carrot (Sekerci et al., 2021), and other species of *Apiaceae*.

The genetic variation of the 31 dill genotypes was examined using both ISSR and SCoT molecular markers, since each marker has a unique aptitude. The effective number of alleles (Ne), I index, and Nei’s unbiased gene diversity mean values were compared, revealing that there were no appreciable differences between the pre-defined populations of landraces and cultivars. According to the I index, there was moderate diversity for both ISSR and SCoT markers (landraces: 0.386, 0.313, cultivars: 0.385, 0.245). Nei’s unbiased haploid gene diversity estimates (uh) were consistent with the observations for the I index, for both markers.

High genetic variation increases the likelihood that populations will be able to adapt to shifting environments. Allelic frequencies are influenced by the survival and progeny rates of various populations. Genetic diversity is reduced through inbreeding, genetic drift, restricted gene flow, and small population size. Accordingly, the polymorphism rates for both molecular markers were high in landraces (76.47% for ISSR and 72.41% for SCoT), and low in cultivars (68.24% for ISSR and 43.1% for SCoT). Thus, the ISSR molecular markers produced more polymorphic bands than SCoT markers, indicating that the ISSRs were more effective in examining molecular diversity in dill genotypes and could be used to create stable heterotic groups between landraces and cultivars. Considering that SCoT markers are known to be linked to functional genes, they may be valuable in tracing patterns of the domestication process, and this could explain the low polymorphism levels observed for SCot markers. If this is true, then the combined analysis of the two marker systems indicates that domesticated genotypes (cultivars) have undergone less selective breeding than landraces but still maintain a high level of genome-wide heterozygosity. Moreover, the PCA analysis for ISSR markers clearly separated some of the cultivars (‘Ambrozja’, ‘Lukullus’, ‘Amat’ and ‘Kronos’) from the landraces (Fig. 3A). Therefore, the use of ISSR markers offers great potential for characterizing closely related dill cultivars, and for assessing the genetic diversity between landraces and modern cultivars.

### Polyphenolic content

Green dill tissues are exploited by the food industry as dried drogue, or raw fresh packed biomass, and/or as biomass supplement in several food products (*e.g*., pickled vegetables, sauces, sour cream, spreadable cheese and dressings). In this respect, it would be beneficial for the food industry to use germplasm with a higher nutritive and medicinal value resulting from the presence of antioxidants, such as polyphenols. It was notable that the Greek local landraces ‘T-538/06’ and ‘GRC-1348/04’ had high amounts of chlorogenic acid (7.972 and 7.997 mg 100 g^−1^ FW, respectively), similar to the those of the modern cultivars ‘Szmaragd’, ‘Ambrozja’, ‘Diana’ and ‘Mariska’ (6.748–8.673 mg 100 g^−1^ FW), indicating the potential of these landraces to be exploited in further breeding programs. Previously, it has been reported that the polyphenol content in the green tissues of dill was dependent on plant height and varied among the plant tissues as follows: leaf blade > petioles > stems (Lisiewska, Kmiecik & Korus, 2006). Based on the morphological traits determined for the dill germplasm in this study, it was observed that ‘T-538/06’ and ‘GRC-1348/04’ scored as ‘tall’ landraces with ‘medium’ to ‘dense’ density of foliage and density of feathering, therefore these morphological descriptors could also be correlated with their higher content in chlorogenic acid. Metabolite levels probably differ in the tissues of different dill plant organs, but such measurements were outside of the scope of this study. Generally, however, it is the fresh, whole plant that is utilized in industrial applications.

### Essential oil content and composition

Variation in essential oil yield was observed for the dill genotypes studied. The yield of the twenty-two Greek landraces ranged from 0.012% to 0.057%, while that of five of the commercial cultivars, ranged from 0.02% to 0.041% (Table S5). Parameters such as, cultivar, seeding date, harvesting stage, and planting density have been shown to affect dill essential oil yield (Biesiada et al., 2019; Bowes et al., 2004; Callan et al., 2007; Kaur, Kaur & Bhardwaj, 2021; Vokk et al., 2011). The leaf essential oil yields of the winter, greenhouse grown dill plants in this study were generally lower than those reported in the literature for dill plants grown in warmer seasons. In a study of fifty-six dill accessions from the US National Plant Germplasm collection, originating from ten different countries, the leaf essential oil contents ranged from 0.1% to 0.3% (mL 100 g^−1^ FW) for plants grown in the spring and harvested in July (Charles, Simon & Widrlechner, 1995). In other studies where yield was expressed as dry weight, leaf essential oil yields of 0.1–0.46% have been reported for four Iranian dill ecotypes harvested in July (Mohebodini & Kalalagh, 2021), of 0.56% for winter-grown and of 0.65% for the leaves of summer-grown dill in Estonia (Vokk et al., 2011).

The main essential oil constituents identified in the dill leaves (and petioles) in this study are in agreement with those reported in the literature for dill herb (leaves and stems): *α*-pinene (0.02–0.12%), *α*-phellandrene (44.44–64.65%), *β*-phellandrene (5.48–19.08%), dill ether (0.94–30.18%), myristicin (0.02–28.18%), dill apiole (0–4.09%) and apiole (1.05–6.92%) (Biesiada et al., 2019; Charles, Simon & Widrlechner, 1995; El-Zaeddi et al., 2020, 2017, 2016; Mohebodini & Kalalagh, 2021; Vokk et al., 2011). However, the content of *α*-phellandrene (61.16–70.25%) in the landrace essential oils was generally higher than values reported in the literature (44.44–64.65%) for leaf essential oil (Biesiada et al., 2019; Charles, Simon & Widrlechner, 1995; El-Zaeddi et al., 2020, 2017, 2016; Mohebodini & Kalalagh, 2021; Vokk et al., 2011).

Dill herb essential oil quality (relative percentage composition of constituents) has been shown to vary with cultivar, seeding date, harvesting stage, irrigation dose, and plant density (Biesiada et al., 2019; Bowes et al., 2004; Callan et al., 2007; El-Zaeddi et al., 2017, 2016; Kaur, Kaur & Bhardwaj, 2021; Vokk et al., 2011). In addition, it differs from that of dill seed, which is characterized by carvone, dihydro-carvone and limonene, and the absence of dill ether (Charles, Simon & Widrlechner, 1995; Vokk et al., 2011). It has been reported that as dill plants mature and flower the content of dill ether in the dill weed (leaves and flowering tops) essential oil decreases, while that of carvone increases (Porter et al., 1983; Bowes et al., 2004; Callan et al., 2007; Vokk et al., 2011).

The aroma of dill leaf and stem essential oil has been mainly attributed to the major constituents, *α*-phellandrene with sensual descriptor ‘dill-like’ ‘fragrant, fresh’ odor and dill ether with sensual descriptor ‘floral, fragrant odor’ with additional ‘green grass’ and ‘citrus’ notes (Blank, Sen & Grosch, 1992; El-Zaeddi et al., 2017, 2016; Pino et al., 1995). Recently however, it has been reported that amino acids and organic acids are also associated with ‘dill’ and ‘sour taste’ (Castro-Alves et al., 2021). Thus, Greek landraces rich in *α*-phellandrene (>68%; ‘T-399/06’, ‘T-208/06’, and ‘T-326/06’) and dill ether (>17.4%; ‘IS-127/07’, ‘KD-235/07’, and ‘T-309/06’) from the current study, could be used in breeding programs for the development of new cultivars with novel aroma quality traits (El-Zaeddi et al., 2017; Lisiewska, Kmiecik & Korus, 2006; Wasli et al., 2018).

## Conclusions

The multivariate analysis of the morphological traits, molecular markers, and secondary metabolites of twenty-two landraces of dill from the Greek Gene Bank collection and nine modern cultivars was successful in evaluating genetic variation among genotypes and in separating various individuals from different populations. In most analyses, even though based on the different evaluator data sets, three main clusters were observed for the 31 genotypes, depicting a clear separation between landraces and modern cultivars. Individual landraces only occasionally clustered with the cultivars in the hierarchical analysis. Some phenotypic variation was evident within the landrace group, but this was more noticeable between the landraces and the cultivars, with the majority of Greek landraces (19 of 22) being classified as ‘tall’, with ‘long’ main stem, ‘large’ main umbel diameter, with ‘many’ peduncles in the umbel, and mainly ‘rhomboidal’ leaf shape.

The ISSR and SCoT molecular markers were effective in describing the genetic diversity between *A. graveolens* genotypes and genetic differences were statistically significant. The pre-defined populations (landraces and modern cultivars) exhibited genetic divergence, but were not entirely isolated from one another, indicating that some amount of gene flow may have occurred between them. Therefore, individuals from pre-defined populations should also be taken into consideration as starting material in genetic improvement programs.

The dill leaf chemical profiles (essential oil and polyphenolic composition) were also effective in discriminating between *A. graveolens* genotypes and in identifying genotypes with ‘particular’ odor and nutritional characteristics. Although some phytochemical variation was determined between the landraces, this was more noticeable between the landraces and the cultivars where, most of the Greek landraces had higher *α*-phellandrene and dill ether contents, associated with the characteristic dill odor, whereas cultivars had higher myristicin and lower dill ether contents. Similarly, polyphenolic composition indicated that some of the landraces (‘T-538/06’ and ‘GRC-1348/04’) were rich in chlorogenic acid, the main polyphenolic compound identified.

In conclusion, this study evaluated simultaneously, and for the first time, the morphological traits, genetic diversity, and chemical profile of dill landraces from a Greek Gene Bank collection, compared to some commercial cultivars. The observed diversity indicated that some landraces could be beneficially used in breeding programs for the development of cultivars, with a particular aroma or nutritional quality, with higher biomass yields, or with earlier harvesting potential, compared to current commercial cultivars. It was also underlined that all the traits, either phytochemical, morphological, agronomic, or genetic, were valuable parameters that should be considered all together in breeding programs.

## Supplemental Information

10.7717/peerj.15043/supp-1 Supplemental Information 1 Phenotypic characterization of germplasm collection based on twenty-two morphological descriptors of UPOV.

10.7717/peerj.15043/supp-2 Supplemental Information 2 Correlation matrix (Spearman) for the evaluation the relationship between the morphological descriptors.

10.7717/peerj.15043/supp-3 Supplemental Information 3 The thirty-one dill genotypes studied (twenty-two Greek landraces and nine commercial cultivars).

10.7717/peerj.15043/supp-4 Supplemental Information 4 The first five components from the PCA analysis of twenty-two morphological descriptors studied for the thirty-one dill genotypes.

10.7717/peerj.15043/supp-5 Supplemental Information 5 Detailed amplification results of each ISSR and SCoT primer used in the study.

10.7717/peerj.15043/supp-6 Supplemental Information 6 Genetic diversity statistics based on ISSR and SCoT markers.

10.7717/peerj.15043/supp-7 Supplemental Information 7 Essential oil composition and content, and concentrations of three polyphenols isolated from the leaves of twenty-two Greek landraces and nine commercial cultivars of dill (*Anethum graveolens* L.).

10.7717/peerj.15043/supp-8 Supplemental Information 8 Agarose gel electrophoresis from PCR reactions using UBC834, UBC811 and SCOT33 primers.

## Additional Information and Declarations

### Data Availability

The following information was supplied regarding data availability: The raw data is available in the Supplemental Files.
