# Supplementary material for: Evaluation of a dill (Anethum graveolens L.) gene bank germplasm collection using multivariate analysis of morphological traits, molecular genotyping and chemical composition to identify novel genotypes for plant breeding
Source: PeerJ. 2023 Mar 29;11:e15043. doi: 10.7717/peerj.15043 (PMC10066692; doi:10.7717/peerj.15043)
Supplement: Supplemental Information 3 [file peerj-11-15043-s003.docx]

**Supplemental Table 1**. The thirty-one dill genotypes studied (twenty-two Greek landraces and nine commercial cultivars).

| **No** | **Genotype** | **Pre-defined population** | **No** | **Genotype** | **Pre-defined population** |
| --- | --- | --- | --- | --- | --- |
| 1 | T-518/06 | Local cultivated Landraces | 23 | Szmaragd | Commercial Cultivars |
| 2 | HL-232/07 |  | 24 | Diana |  |
| 3 | T-208/06 |  | 25 | Mariska |  |
| 4 | T-538/06 |  | 26 | Tetra |  |
| 5 | T-370/06 |  | 27 | Dukat |  |
| 6 | T-349/06 |  | 28 | Kronos |  |
| 7 | GRC-209/08 |  | 29 | Ambrozja |  |
| 8 | GRC-1348/04 |  | 30 | Lukullus |  |
| 9 | IS-127/07 |  | 31 | Amat |  |
| 10 | T-326/06 |  |  |  |  |
| 11 | T-315/06 |  |  |  |  |
| 12 | T-309/06 |  |  |  |  |
| 13 | T-269/06 |  |  |  |  |
| 14 | KD-178/07 |  |  |  |  |
| 15 | XKA-035/07 |  |  |  |  |
| 16 | T-382/06 |  |  |  |  |
| 17 | ROX-064/07 |  |  |  |  |
| 18 | ANP-015/07 |  |  |  |  |
| 19 | T-399/06 |  |  |  |  |
| 20 | KD-235/07 |  |  |  |  |
| 21 | SAS-049/07 |  |  |  |  |
| 22 | K-133/06 |  |  |  |  |
