# Supplementary material for: Evaluation of a dill (Anethum graveolens L.) gene bank germplasm collection using multivariate analysis of morphological traits, molecular genotyping and chemical composition to identify novel genotypes for plant breeding
Source: PeerJ. 2023 Mar 29;11:e15043. doi: 10.7717/peerj.15043 (PMC10066692; doi:10.7717/peerj.15043)
Supplement: Supplemental Information 4 [file peerj-11-15043-s004.docx]

**Table S2** The first five components from the PCA analysis of twenty-two morphological descriptors studied for the thirty-one dill genotypes.

| **Accession** | ***F1*** | ***F2*** | ***F3*** | ***F4*** | ***F5*** |
| --- | --- | --- | --- | --- | --- |
| Young plant: anthocyanin coloration (YPAC) | 0,238 | -0,658 | 0,329 | -0,223 | -0,154 |
| Young plant: attitude of leaves (YPAL) | 0,381 | 0,462 | 0,018 | 0,623 | -0,073 |
| Plant: Density of foliage (PDF) | 0,119 | 0,165 | 0,432 | 0,376 | 0,341 |
| Plant: Number of primary branches (PNPB) | -0,641 | 0,071 | 0,540 | 0,040 | 0,091 |
| Plant: height (PH) | -0,701 | -0,250 | -0,252 | 0,192 | -0,210 |
| Plant: Length of main stem (PLMS) | -0,722 | -0,296 | -0,180 | 0,194 | -0,255 |
| Stem: diameter (SD) | -0,390 | 0,125 | 0,603 | -0,135 | -0,235 |
| Stem: Blue hue on stem (SBH) | 0,492 | -0,120 | -0,042 | -0,429 | 0,367 |
| Stem: Intensity of green color on stem (SIG) | 0,594 | 0,111 | 0,324 | 0,093 | -0,041 |
| Stem: waxiness (SWAX) | 0,469 | -0,018 | 0,313 | -0,287 | -0,253 |
| Leaf: shape(LS) | -0,612 | -0,537 | 0,079 | -0,024 | -0,086 |
| Leaf: Density of feathering (LDF) | -0,372 | 0,090 | 0,395 | 0,386 | 0,637 |
| Leaf: width of segments (LWS) | -0,296 | 0,524 | -0,256 | -0,282 | -0,012 |
| Leaf: length (LL) | -0,122 | 0,329 | 0,037 | 0,159 | -0,256 |
| Leaf: width (LW) | -0,231 | 0,724 | 0,158 | -0,132 | -0,162 |
| Leaf: Blue hue (LBH) | 0,888 | 0,146 | -0,184 | 0,094 | 0,153 |
| Leaf: Intensity of green color (LIG) | 0,355 | 0,524 | 0,269 | -0,079 | -0,512 |
| Leaf: waxiness (LWAX) | 0,627 | -0,165 | 0,199 | 0,154 | -0,236 |
| Umbel: main umbel diameter (UD) | -0,292 | 0,044 | 0,608 | -0,397 | 0,139 |
| Umbel: number of peduncles (UNP) | 0,034 | 0,116 | -0,180 | -0,480 | 0,321 |
| Umbel: time of appearance of main umbel (UTAMU) | 0,326 | -0,614 | 0,209 | 0,126 | -0,070 |
| Umbel: time of beginning of flowering (UTBF) | 0,374 | -0,714 | 0,102 | 0,136 | -0,054 |
| Eigenvalue | 4,916 | 3,300 | 2,108 | 1,681 | 1,495 |
| Variability (%) | 22,348 | 14,999 | 9,581 | 7,642 | 6,794 |
| Cumulative % | 22,348 | 37,346 | 46,927 | 54,569 | 61,362 |
