# Supplementary material for: Evaluation of a dill (Anethum graveolens L.) gene bank germplasm collection using multivariate analysis of morphological traits, molecular genotyping and chemical composition to identify novel genotypes for plant breeding
Source: PeerJ. 2023 Mar 29;11:e15043. doi: 10.7717/peerj.15043 (PMC10066692; doi:10.7717/peerj.15043)
Supplement: Supplemental Information 5 [file peerj-11-15043-s005.docx]

**Table S3**. Detailed amplification results of each ISSR and SCoT primer used in the study.

| **Primer ID** | **NPB^a^** | | **TNB^b^** | | **PB^c^ (%)** | | **PIC^d^** | **MI^e^** | | **Rp** | |
| --- | --- | --- | --- | --- | --- | --- | --- | --- | --- | --- | --- |
| **ISSR** |  |  | |  | |  | | |  | |  |
| UBC807 | 13 | 17 | | 76.47 | | 0.296 | | | 0.341 | | 5.921 |
| UBC810 | 8 | 13 | | 61.54 | | 0.282 | | | 0.320 | | 5.399 |
| UBC811 | 6 | 10 | | 60.00 | | 0.281 | | | 0.289 | | 4.932 |
| UBC834 | 10 | 14 | | 71.43 | | 0.345 | | | 0.396 | | 7.832 |
| UBC840 | 16 | 21 | | 76.19 | | 0.303 | | | 0.332 | | 7.565 |
| UBC860 | 7 | 10 | | 70.00 | | 0.330 | | | 0.389 | | 6.445 |
| Mean | 10 | 14.16 | | 69.27 | | 0.306 | | | 0.344 | | 6.349 |
| **SCoT** |  |  | |  | |  | | |  | |  |
| SCoT1 | 8 | 14 | | 57.14 | | 0.259 | | | 0.268 | | 4.845 |
| SCoT13 | 7 | 10 | | 70.00 | | 0.331 | | | 0.339 | | 7.654 |
| SCoT33 | 10 | 13 | | 76.92 | | 0.227 | | | 0.299 | | 7.236 |
| SCoT34 | 7 | 11 | | 63.64 | | 0.163 | | | 0.172 | | 6.832 |
| SCoT51 | 4 | 10 | | 40.00 | | 0.092 | | | 0.101 | | 4.747 |
| SCoT61 | 5 | 11 | | 45.45 | | 0.170 | | | 0.185 | | 5.112 |
| Mean | 6.83 | 11.5 | | 58.85 | | 0.207 | | | 0.227 | | 6.071 |

*^a^ NPB: number of polymorphic bands; ^b^ TNB: total number of bands; ^c^ PB: polymorphic band percentage; ^d^ PIC: polymorphism information content, ^e^ MI: marker index, ^f^ Rp:* *resolving power.*
