# Supplementary material for: Evaluation of a dill (Anethum graveolens L.) gene bank germplasm collection using multivariate analysis of morphological traits, molecular genotyping and chemical composition to identify novel genotypes for plant breeding
Source: PeerJ. 2023 Mar 29;11:e15043. doi: 10.7717/peerj.15043 (PMC10066692; doi:10.7717/peerj.15043)
Supplement: Supplemental Information 6 [file peerj-11-15043-s006.docx]

**Table S4.** Genetic diversity statistics based on ISSR and SCoT markers.

|  | ***N^a^*** | **ISSR**  **ne^b^** | ***I^c^*** | ***uh^d^*** | ***P ^e^(%)*** | ***Nei D^f^*** | **Group** |  | ***N*** | **SCOT ne** | ***I*** | ***uh*** | ***P (%)*** | ***Nei D*** |
| --- | --- | --- | --- | --- | --- | --- | --- | --- | --- | --- | --- | --- | --- | --- |
| Mean | 22 | 1.437 | 0.386 | 0.269 | 76.47 |  | Landrace | Mean | 22 | 1.315 | 0.313 | 0.208 | 72.41 |  |
| SE |  | 0.040 | 0.029 | 0.022 |  |  |  | SE |  | 0.042 | 0.032 | 0.024 |  |  |
| Mean | 9 | 1.463 | 0.385 | 0.295 | 68.24 |  | Cultivar | Mean | 9 | 1.299 | 0.245 | 0.188 | 43.1 |  |
| SE |  | 0.043 | 0.031 | 0.025 |  |  |  | SE |  | 0.053 | 0.039 | 0.031 |  |  |
| Mean | 31 | 1.450 | 0.386 | 0.282 | 72.35 | 0.111 | All | Mean | 31 | 1.307 | 0.279 | 0.198 | 57.76 | 0.057 |
| SE |  | 0.029 | 0.021 | 0.017 |  |  |  | SE |  | 0.034 | 0.025 | 0.019 |  |  |

*^a^ N: sample size, ^b^ ne: Number of effective alleles, ^c^ I: Shannon’s Information Index, ^d^ uh: Nei’s unbiased haploid gene diversity, ^e^ P (%): Percent of Polymorphism, ^f^ Nei D: Nei’s Distance 1978.*
