# Supplementary material for: Evaluation of a dill (Anethum graveolens L.) gene bank germplasm collection using multivariate analysis of morphological traits, molecular genotyping and chemical composition to identify novel genotypes for plant breeding
Source: PeerJ. 2023 Mar 29;11:e15043. doi: 10.7717/peerj.15043 (PMC10066692; doi:10.7717/peerj.15043)
Supplement: Supplemental Information 7 [file peerj-11-15043-s007.docx]

**Supplemental Table 5.** Essential oil composition and content, and concentrations of three polyphenols isolated from the leaves of twenty-two Greek landraces and nine commercial cultivars of dill (Anethum graveolens L.).

| **Pre-defined population** |  |  | **Landraces** | | | | | | | | | | | | | | | | | | | | | | | **Cultivars** | | | | | | | | |
| --- | --- | --- | --- | --- | --- | --- | --- | --- | --- | --- | --- | --- | --- | --- | --- | --- | --- | --- | --- | --- | --- | --- | --- | --- | --- | --- | --- | --- | --- | --- | --- | --- | --- | --- |
|  |  | **Arithmetic Index** | **T-518/06** | **HL-232/07** | **T-208/06** | **T-538/06** | **T-370/06** | **T-349/06** | **GRC-209/08** | **GRC-1348/04** | **IS-127-07** | **T-326/06** | **T-315/06** | **T-309/06** | **T-269/06** | **KD-178/07** | **CHA-35/07** | **T-382/06** | **RΟΧ-64/07** | **ΑΝP-15/07** | **Τ-399/06** | **KD-235/07** | **SAS-49/07** | **K-133/06** | **Szmaragd** | | **Diana** | **Mariska** | **Tetra** | **Dukat** | **Kronos** | **Ambrozja** | **Lukullus** | **Amat** |
|  |  |  | **Area Percent (%)** | | | | | | | | | | | | | | | | | | | | | | | | | | | | | | | |
| **Secondary Metabolite** | ***α*-thujene** | 925 | 0.27 | 0.26 | 0.27 | 0.26 | 0.26 | 0.24 | 0.24 | 0.25 | 0.22 | 0.27 | 0.27 | 0.22 | 0.27 | 0.27 | 0.26 | 0.26 | 0.27 | 0.27 | 0.27 | 0.27 | 0.27 | 0.25 | 0.25 | | 0.25 | 0.22 | 0.22 | 0.21 | 0.43 | 0.44 | 0.45 | 0.44 |
|  | ***α*-pinene** | 932 | 1.38 | 1.32 | 1.33 | 1.31 | 1.33 | 1.24 | 1.18 | 1.23 | 1.09 | 1.38 | 1.31 | 1.07 | 1.28 | 1.34 | 1.26 | 1.26 | 1.35 | 1.36 | 1.34 | 1.30 | 1.38 | 1.23 | 1.21 | | 1.14 | 1.06 | 1.08 | 0.99 | 2.22 | 2.30 | 2.33 | 2.27 |
|  | **sabinene** | 972 | 0.11 | 0.12 | 0.11 | 0.11 | 0.10 | 0.09 | 0.09 | 0.09 | 0.10 | 0.12 | 0.11 | 0.11 | 0.10 | 0.12 | 0.11 | 0.10 | 0.11 | 0.10 | 0.11 | 0.11 | 0.10 | 0.09 | 0.08 | | 0.10 | 0.07 | 0.08 | 0.07 | 0.20 | 0.20 | 0.22 | 0.21 |
|  | ***β*-pinene** | 976 | 0.26 | 0.12 | 0.23 | 0.22 | 0.29 | 0.27 | 0.43 | 0.39 | 0.11 | 0.17 | 0.23 | 0.12 | 0.34 | 0.20 | 0.44 | 0.25 | 0.12 | 0.24 | 0.25 | 0.13 | 0.26 | 0.44 | 0.38 | | 0.13 | 0.29 | 0.17 | 0.44 | 0.11 | 0.16 | 0.16 | 0.13 |
|  | **myrcene** | 992 | 0.68 | 0.65 | 0.68 | 0.69 | 0.68 | 0.66 | 0.67 | 0.65 | 0.61 | 0.69 | 0.70 | 0.63 | 0.67 | 0.70 | 0.68 | 0.66 | 0.66 | 0.70 | 0.73 | 0.67 | 0.72 | 0.74 | 0.66 | | 0.66 | 0.60 | 0.59 | 0.61 | 0.91 | 0.89 | 0.91 | 0.90 |
|  | ***α*-phellandrene** | 1009 | 64.28 | 65.41 | 68.45 | 66.35 | 67.83 | 65.49 | 63.56 | 63.73 | 61.16 | 68.02 | 67.81 | 63.94 | 65.65 | 67.10 | 65.65 | 63.92 | 64.43 | 66.66 | 70.25 | 65.16 | 67.27 | 67.47 | 62.03 | | 62.31 | 54.42 | 56.12 | 55.14 | 59.95 | 61.79 | 63.86 | 63.56 |
|  | ***α*-terpinene** | 1010 | 0.16 | 0.00 | 0.08 | 0.14 | 0.00 | 0.39 | 0.35 | 0.30 | 0.00 | 0.07 | 0.16 | 0.00 | 0.10 | 0.06 | 0.03 | 0.17 | 0.00 | 0.00 | 0.00 | 0.00 | 0.13 | 0.00 | 0.09 | | 0.00 | 0.00 | 0.01 | 0.00 | 0.02 | 0.02 | 0.02 | 0.02 |
|  | ***p*-cymene** | 1017 | 0.12 | 0.12 | 0.13 | 0.12 | 0.12 | 0.12 | 0.12 | 0.12 | 0.11 | 0.12 | 0.13 | 0.12 | 0.12 | 0.12 | 0.11 | 0.12 | 0.12 | 0.12 | 0.13 | 0.12 | 0.13 | 0.13 | 0.11 | | 0.11 | 0.09 | 0.10 | 0.09 | 0.11 | 0.12 | 0.12 | 0.12 |
|  | **limonene** | 1025 | 1.37 | 0.61 | 0.67 | 0.68 | 0.73 | 0.67 | 0.80 | 0.80 | 0.74 | 0.75 | 0.59 | 0.77 | 0.69 | 0.69 | 0.64 | 0.61 | 0.55 | 0.63 | 0.77 | 0.66 | 0.71 | 0.86 | 0.79 | | 1.10 | 1.00 | 1.05 | 1.15 | 1.65 | 2.02 | 0.98 | 1.50 |
|  | ***β*-phellandrene** | 1029 | 12.13 | 11.00 | 11.69 | 11.52 | 11.51 | 11.47 | 10.76 | 10.77 | 10.62 | 11.68 | 11.68 | 11.41 | 11.06 | 11.34 | 11.13 | 10.87 | 10.70 | 11.13 | 12.35 | 10.98 | 11.46 | 11.72 | 10.59 | | 10.95 | 9.38 | 9.52 | 9.72 | 11.88 | 11.98 | 12.08 | 12.05 |
|  | **(Z)-*β*-ocimene** | 1038 | 0.02 | 0.02 | 0.02 | 0.02 | 0.00 | 0.02 | 0.03 | 0.03 | 0.02 | 0.00 | 0.02 | 0.01 | 0.02 | 0.02 | 0.03 | 0.02 | 0.04 | 0.05 | 0.02 | 0.03 | 0.05 | 0.03 | 0.11 | | 0.08 | 0.11 | 0.09 | 0.12 | 0.02 | 0.02 | 0.02 | 0.02 |
|  | **(E)-*β*-ocimene** | 1048 | 0.18 | 0.23 | 0.13 | 0.10 | 0.13 | 0.16 | 0.12 | 0.16 | 0.04 | 0.13 | 0.05 | 0.09 | 0.31 | 0.05 | 0.09 | 0.08 | 0.14 | 0.25 | 0.07 | 0.06 | 0.22 | 0.09 | 0.64 | | 0.44 | 0.63 | 0.56 | 0.62 | 0.03 | 0.03 | 0.03 | 0.03 |
|  | **terpinolene** | 1088 | 0.23 | 0.18 | 0.23 | 0.17 | 0.18 | 0.19 | 0.16 | 0.20 | 0.14 | 0.17 | 0.20 | 0.15 | 0.17 | 0.17 | 0.16 | 0.17 | 0.16 | 0.18 | 0.16 | 0.16 | 0.17 | 0.24 | 0.14 | | 0.14 | 0.12 | 0.11 | 0.12 | 0.22 | 0.21 | 0.24 | 0.22 |
|  | **linalool** | 1100 | 0.28 | 0.37 | 0.31 | 0.86 | 0.50 | 1.61 | 2.06 | 1.98 | 0.16 | 0.46 | 0.44 | 0.17 | 1.04 | 0.16 | 0.56 | 0.51 | 1.63 | 0.99 | 0.49 | 0.32 | 0.83 | 0.45 | 1.71 | | 0.88 | 1.17 | 0.87 | 1.51 | 0.33 | 0.17 | 0.18 | 0.21 |
|  | **dill ether** | 1188 | 15.30 | 15.48 | 13.57 | 14.98 | 13.90 | 10.67 | 4.81 | 12.73 | 22.22 | 13.85 | 13.34 | 17.41 | 14.26 | 14.87 | 9.63 | 15.50 | 4.48 | 5.04 | 10.47 | 17.51 | 7.93 | 8.72 | 0.86 | | 2.69 | 0.66 | 0.77 | 0.62 | 4.61 | 12.13 | 10.03 | 7.88 |
|  | **carvacrol** | 1299 | 0.01 | 0.11 | 0.08 | 0.08 | 0.08 | 0.17 | 0.29 | 0.17 | 0.08 | 0.08 | 0.10 | 0.08 | 0.13 | 0.07 | 0.12 | 0.13 | 0.23 | 0.14 | 0.07 | 0.08 | 0.16 | 0.13 | 0.25 | | 0.11 | 0.16 | 0.11 | 0.23 | 0.30 | 0.48 | 0.11 | 0.25 |
|  | **germacrene D** | 1479 | 0.26 | 0.92 | 0.64 | 0.53 | 0.60 | 0.76 | 2.14 | 0.58 | 0.54 | 0.79 | 0.23 | 0.91 | 0.75 | 0.60 | 0.40 | 0.95 | 0.77 | 0.23 | 0.36 | 0.57 | 0.16 | 0.76 | 0.47 | | 0.32 | 0.72 | 0.73 | 0.64 | 0.05 | 0.07 | 0.05 | 0.12 |
|  | **myristicin** | 1524 | 0.00 | 1.75 | 0.00 | 0.00 | 0.00 | 0.05 | 4.26 | 0.02 | 0.01 | 0.00 | 0.00 | 0.01 | 0.00 | 0.02 | 7.81 | 0.94 | 10.82 | 7.11 | 0.02 | 0.02 | 7.23 | 4.97 | 13.60 | | 15.18 | 16.63 | 18.56 | 16.19 | 14.39 | 4.20 | 6.71 | 6.23 |
|  | **dill apiole** | 1633 | 1.74 | 0.09 | 0.40 | 0.73 | 0.86 | 4.05 | 5.88 | 3.85 | 0.48 | 0.29 | 1.32 | 0.47 | 1.77 | 0.88 | 0.01 | 1.45 | 2.02 | 3.86 | 1.16 | 0.11 | 0.01 | 0.02 | 4.98 | | 2.44 | 11.12 | 7.41 | 10.00 | 0.76 | 0.75 | 0.15 | 2.23 |
|  | ***α*-cadinol** | 1660 | 0.11 | 0.26 | 0.12 | 0.18 | 0.22 | 0.27 | 0.41 | 0.56 | 0.23 | 0.24 | 0.21 | 0.21 | 0.34 | 0.21 | 0.27 | 0.37 | 0.25 | 0.14 | 0.16 | 0.26 | 0.05 | 0.42 | 0.14 | | 0.10 | 0.20 | 0.20 | 0.16 | 0.00 | 0.01 | 0.00 | 0.01 |
|  | **apiole** | 1689 | 0.00 | 0.02 | 0.00 | 0.00 | 0.00 | 0.00 | 0.24 | 0.01 | 0.00 | 0.00 | 0.00 | 0.00 | 0.00 | 0.00 | 0.09 | 0.00 | 0.29 | 0.07 | 0.00 | 0.00 | 0.02 | 0.04 | 0.09 | | 0.08 | 0.29 | 0.43 | 0.23 | 0.43 | 0.10 | 0.16 | 0.14 |
| **Essential oil content (%)** |  |  | 0,032 | 0,057 | 0,037 | 0,025 | 0,035 | 0,024 | 0,012 | 0,027 | 0,020 | 0,030 | 0,037 | 0,016 | 0,034 | 0,024 | 0,038 | 0,049 | 0,040 | 0,029 | 0,027 | 0,026 | 0,045 | 0,019 | 0,032 | | 0,041 | 0,035 | 0,023 | 0,020 | nd^1^ | nd | nd | nd |
| **Polyphenols (mg /100 g FW)** | **neo-chlorogenic acid** |  | 0.0032 | 0.0002 | 0.0011 | 0.0016 | 0.0009 | 0.0011 | 0.0016 | 0.0000 | 0.0147 | 0.0004 | 0.0000 | 0.0008 | 0.0008 | 0.0005 | 0.0000 | 0.0003 | 0.0009 | 0.0008 | 0.0008 | 0.0005 | 0.0003 | 0.0011 | 0.0013 | | 0.0010 | 0.0017 | 0.0022 | 0.0018 | 0.0016 | 0.0012 | 0.0015 | 0.0015 |
|  | **Chlorogenic acid** |  | 5.7072 | 6.1133 | 3.6699 | 7.9720 | 4.8326 | 4.2894 | 3.9753 | 7.9970 | 6.3506 | 2.1198 | 4.2379 | 5.5182 | 5.8662 | 2.0354 | 4.9507 | 3.9046 | 6.0154 | 5.5547 | 5.3678 | 4.3671 | 5.1269 | 4.9862 | 7.2130 | | 6.7480 | 8.6727 | 10.3503 | 9.7525 | 6.5218 | 7.8642 | 6.5876 | 6.9851 |
|  | **Quercetin-3-glucuronide** |  | 0.2448 | 0.1057 | 0.0035 | 0.1053 | 0.1105 | 0.09152 | 0.1145 | 0.0092 | 0.1152 | 0.2251 | 0.0767 | 0.1643 | 0.1404 | 0.1636 | 0.1911 | 0.2604 | 0.1246 | 0.1058 | 0.0954 | 0.1288 | 0.1158 | 0.1511 | 0.3547 | | 0.3574 | 0.3301 | 0.4351 | 0.3063 | 0.3197 | 0.3234 | 0.3125 | 0.3412 |
|  | **Polyphenol SUM** |  | 5.955 | 6.219 | 3.675 | 8.079 | 4.944 | 4.382 | 4.091 | 8.006 | 6.481 | 2.345 | 4.315 | 5.683 | 6.007 | 2.199 | 5.142 | 4.165 | 6.141 | 5.661 | 5.464 | 4.496 | 5.243 | 5.138 | 7.569 | | 7.106 | 9.004 | 10.788 | 10.061 | 6.843 | 8.189 | 6.902 | 7.328 |

^1^nd, not determined
