# Supplementary figures and images for: Evaluation of a dill (Anethum graveolens L.) gene bank germplasm collection using multivariate analysis of morphological traits, molecular genotyping and chemical composition to identify novel genotypes for plant breeding
Source: PeerJ. 2023 Mar 29;11:e15043. doi: 10.7717/peerj.15043 (PMC10066692; doi:10.7717/peerj.15043)

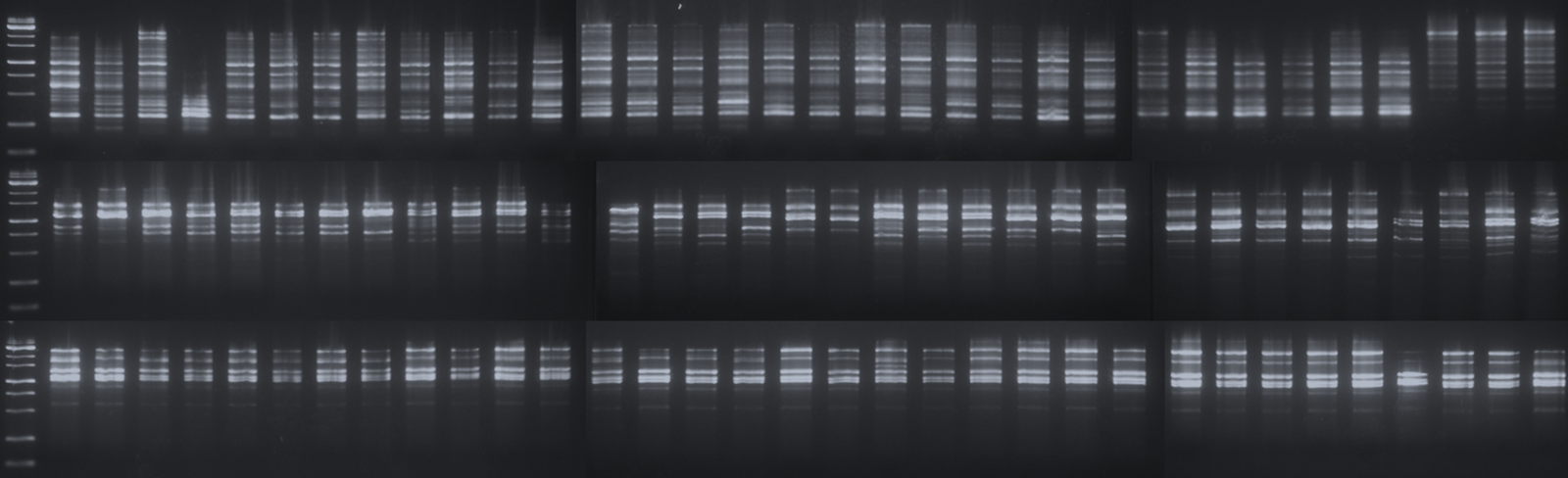

Supplement: Supplemental Information 8 [file peerj-11-15043-s008.png]
